# A study of HER2 expression in endometrial carcinoma: a single centre experience

**DOI:** 10.11604/pamj.2021.38.200.19978

**Published:** 2021-02-22

**Authors:** Mariani Hashim, Nur Maya Sabrina Tizen, Nurwardah Alfian, Hasmah Hashim, Azmawati Mohammed Nawi, Suria Hayati Md Pauzi

**Affiliations:** 1 Department of Pathology, Hospital Canselor Tuanku Mukhriz, Universiti Kebangsaan, Kuala Lumpur, Malaysia,; 2 Department of Pathology, Hospital Melaka, Melaka, Malaysia,; 3 Department of Community Health, Hospital Canselor Tuanku Mukhriz, Universiti Kebangsaan, Kuala Lumpur, Malaysia

**Keywords:** HER2, endometrial carcinoma, endometrioid carcinoma

## Abstract

**Introduction:**

endometrial carcinoma (EC) is the seventh most common cancer in females in Malaysia, of which the majority is composed of lower grade type I EC. Although less prevalent, type II EC which is of higher grade has poorer outcome and prognosis. Human epidermal growth factor receptor 2 (HER2) is one of the possible prognostic molecular markers which can be a target for immunotherapy. This study aimed to assess the expression of HER2 in common type of EC in the local population and to determine its correlation with the clinicopathological features.

**Methods:**

a total of 53 cases of endometrioid type of EC were selected within a six-year period comprising of 22 cases of grade 1, 25 cases of grade 2 and six cases of grade 3 carcinoma. The selected whole tumour tissue sections were immune-stained with HER2 antibody. The scoring was semi-quantitatively analyzed based on 2013 American Society of Clinical Oncology (ASCO)/College of American Pathologists (CAPs) guidelines for the scoring of HER2 in breast cancer.

**Results:**

all cases regardless of grades of endometrioid carcinoma showed negative expression of HER2 (score 0).

**Conclusion:**

there was no significant HER2 expression in endometrioid carcinoma. However, a follow-up study with a larger number of samples from different type of endometrial carcinoma is needed. Testing of several tumour tissue blocks to assess possible tumour heterogeneity, as well as correlation with HER2 gene amplification status by in-situ-hybridisation, are also recommended.

## Introduction

Endometrial carcinoma (EC) is the second most common gynecological malignancy worldwide [1] with an estimated 52,630 new cases and 8,590 death in the United States of America alone in the year 2014 [2]. In Malaysia, EC was the seventh most common cancer in females [3]. EC is categorized into type I and type II tumour. The majority of tumours (approximately 80-90%) are classified as type I EC comprises of endometrioid carcinoma and its variants as well as mucinous carcinoma [3]. Serous and clear cell carcinomas, on the other hand, are the prototypically type II tumour. Nevertheless, in a few studies, grade 3 endometrioid carcinoma is also considered as type II EC [4, 5]. The pathogenesis of type I EC is related to longer duration of oestrogen exposure such as earlier age of menarche, later age at menopause, nulliparity and obesity [6]. As compared to type II EC, an association of this more aggressive type of tumour with multiparity, postmenopausal status, cigarette smoking, history of breast carcinoma and/or tamoxifen use are more commonly observed [6].

EC is staged pathologically according to WHO based on American Joint Committee on Cancer TNM staging and International Federation of Gynecology and Obstetrics (FIGO) staging which classify the tumour on the basis of three factors which is the extent of the tumour (T), lymph node spread (N) and distant metastasis (M) [7]. While all variant of type II tumour is considered as high grade, grading of endometrioid carcinoma are assigned primarily based on their architecture. Grade 1 is considered when the tumour has 5% or less solid growth, those between 6-50% and more than 50% solid growth are classified as grade 2 and grade 3, respectively. The presence of grade 3 nuclei involving greater than 50% of tumour is associated with more aggressive behaviour, and therefore justifies upgrading the tumour by one grade [6]. These pathological features of the tumour which include grade and stage are associated with prognosis and predictive outcomes of EC. The overall five-year survival rates for EC are approximately 78-90% for stage I, 74% for stage II, 36-57% for stage III, and 20% for stage IV [8, 9]. Additionally, women with metastatic disease have a median survival of seven to 12 months only [10]. Such poor outcomes raise an urgent requirement for more accurate prognostic and predictive markers for EC to help in guiding the therapy and monitor the disease´s progress for individual patients.

The recent and ongoing researches in molecular pathways of EC have led to the identification of prognostic molecular markers, among which human epidermal growth factor receptor 2 (HER2) is of interest. HER2 (ErbB2) is a member of the human epidermal growth factor receptor (EGFR), a family of transmembrane tyrosine kinases. The other member including EGFR (HER1, ErbB1), HER2/neu (ErbB2), HER3 (ErbB3) and HER4 (ErbB4). HER2 overexpression results in ligand-independent dimer formation and constitutive activation of the kinase domain, leading to an increase in cell proliferation [11]. HER2 amplification and overexpression have been shown to play a key role in the pathogenesis of various cancer types including breast, ovarian, gastric, oesophageal carcinoma and endometrial carcinoma [4]. To our knowledge, there are no published data reporting on HER2 expression in EC in Malaysia. This study aimed to give an overview of EC seen in a single medical centre over a six-year period (2012-2017) and to assess the expression of HER2 protein by immuno histochemistry and its possible correlation with the clinicopathological features. The potential use of HER2 in routine pathological service as one of the prognostic markers was evaluated in this study.

## Methods

**Ethical approval:** this study was approved by the Ethical Committee of Universiti Kebangsaan Malaysia (reference no: UKM PPI/111/8/JEP-2017-121, Project code: FF-2017-094).

**Tissue samples:** a total of 53 cases of endometrial carcinoma from 2012 to 2017 were retrieved from the archives of Histopathology Unit, Department of Pathology, Hospital Melaka, Malaysia. Only hysterectomy specimens were included in this study while cases diagnosed from the pipelle sampling or endometrial curettage were excluded. Clinicopathological parameters including age, ethnic, parity, menopausal status, tumour type, tumour grade, myometrial invasion and tumour stage were reviewed from patient´s medical records. The slides from the selected cases were reviewed and one tumour tissue block was selected from each case for immunohistochemistry study.

**Immunohistochemical studies:** a three-micro meter thick section from each selected formalin-fixed, paraffin-embedded tissue block was cut and mounted onto coated slides. HER2 immunohisto chemistry (IHC) was performed using DAKO anti-HER2/neu (Code A0485, rabbit polyclonal; pre-dilution; Dako Denmark) antibody and its detection kit (EnVision FLEX Mini Kit High pH (Dako AS Plus) on an automatic immunostainer (BenchMark XT, Ventana Medical Systems), according to the manufacturer´s instructions. Primary antibody was omitted for negative control. Breast cancer tissues with different scoring (3+, 2+ and 1+/0) were used as the positive control tissue.

**Immunohistochemical analysis:** IHC scoring was independently performed by two observers without prior knowledge of the clinicopathological information. Whenever there was a discordant result, the slides were reviewed together, and a consensus was agreed upon. Positive HER2 staining is membranous. The scoring was semi-quantitatively analyzed based on 2013 American Society of Clinical Oncology (ASCO)/College of American Pathologists (CAPs) guidelines for scoring of HER2 in breast cancer (ASCO/CAP 2013) [12]. The four categories of scoring were as follows: score 0 if no staining; score 1+ showing incomplete membrane staining that is faint/barely perceptible in >10% of tumour cells; score 2+ showing incomplete and/or weak/moderate membrane staining in >10% of tumour cells and score 3+ exhibiting circumferential, intense, complete membrane staining >10% of tumour cells. IHC score 0 or 1+ is considered negative for HER2 expression while IHC 2+ and 3+ will be taken as HER2-IHC positive group for statistical analysis.

**Statistics:** all data collected were tabulated accordingly and analyzed using Statistical Packaged for the Social Science, (SPSS) software version 23. The result was analyzed statistically by the descriptive percentage of frequency.

## Results

A total of 53 EC patients, who fulfilled the inclusion criteria were included in this study. The youngest age at diagnosis was 29 years (patient age ranged from 29 - 75 years, mean = 55.5). The majority of cases were seen among Malay ethnic (n = 36, 67.9%) followed by Chinese (n = 11, 20.8%) and Indian (n = 6, 11.3%). EC is more frequently seen in parous women (n = 36, 67.9%) than nullipara (n = 17, 32.1%). The incidence among pre-menopausal and post-menopausal women was almost similar with the frequency of 26 (49.1%) and 27 (50.9%), respectively (Table 1).

**Table 1:** clinicopathologic variables of the study population

Variables	No. of cases (%)
**Ethnic**	
Malay	36 (67.9%)
Chinese	11 (20.8%)
Indian	6 (11.3%)
**Parity**	
Nullipara	17 (32.1%)
Multipara	36 (67.9%)
**Menopausal status**	
Pre-menopause	26 (49.1%)
Post-menopause	27 (50.9%)
**Tumour type**	
Type 1 EC	47 (88.7%)
Type 2 EC (grade 3 endometrioid carcinoma)	6 (11.3%)
**Tumour grade (Endometrioid carcinoma)**	
Grade 1	22 (41.5%)
Grade 2	25 (47.2%)
Grade 3	6 (11.3%)
**Myometrial invasion**	
Less than 50%	26 (49.1%)
More than 50%	27 (50.9%)
**pT stage**	
T1	41 (77.4%)
T2	3 (5.7%)
T3	7 (13.2%)
T4	2 (3.7%)
**Metastasis**	
Absent	51 (96.2%)
Present	2 (3.8%)

All the cases encountered were endometrioid carcinoma. None of the serous or clear cell carcinoma was obtained over the period of study. Most of the cases were categorized as type 1 EC (n = 47, 88.7%), out of which 22 cases (41.5%) were endometrioid carcinoma grade 1 and 25 cases (47.2%) were endometrioid carcinoma grade 2. There were only six cases categorized as type 2 EC (11.3%) which were endometrioid carcinoma grade 3 (Table 1).

A total of 26 cases showed tumour invasion occupying less than 50% of myometrial thickness (49.1%) whereby 27 cases exhibited extensive myometrial infiltration of more than 50% of myometrial thickness (50.9%). A total of 41 cases (77.4%) were staged as FIGO stage 1, three cases (5.7%) were at stage 2 disease, seven cases (13.2%) were at stage 3 and two cases (3.8%) were categorized as stage 4 (Table 1). The two cases of stage 4 EC had distant metastases to bladder and small bowel, respectively.

**Figure 1:**
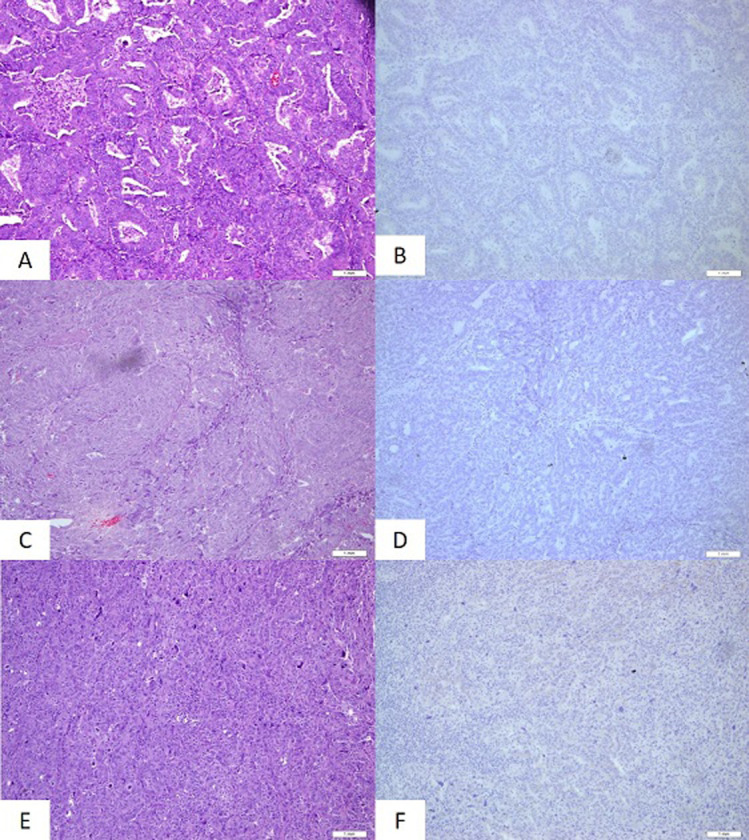
A) endometrioid carcinoma, grade 1 (H&E, 100x magnification, scale bar = 1mm); B) endometrioid carcinoma, grade 1 with negative HER2 expression (100x magnification, scale bar = 1mm); C) endometrioid carcinoma, grade 2 (H&E, 100x magnification, scale bar = 1mm); D) endometrioid carcinoma, grade 2 with negative HER2 expression (100x magnification, scale bar = 1mm); E) endometrioid carcinoma, grade 3 (H&E, 100x magnification, scale bar = 1mm); F) endometrioid carcinoma, grade 3 with negative HER2 expression) (100x magnification, scale bar = 1mm)

**HER2 protein expression by immunohisto chemistry:** immunohistochemistry studies were performed to tumour tissue sections from the selected cases comprising of endometrioid tumour with grade 1 (n = 22, 41.5%), grade 2 endometroid (n = 25, 47.2%) and grade 3 (n = 6, 11.3%). All the cases showed negative HER2 expression (score 0) regardless of tumour grade (Table 2, Figure 1). The control tissue which comprised of tumour with negative (0 and 1+), equivocal (2+) and positive (3+) were working well with good staining.

**Table 2:** HER2 protein expression in endometrial carcinoma (EC) by immunohistochemistry (IHC)

Grade of EC	0	1+	2+	3+
Grade 1	22 (41.5%)	0	0	0
Grade 2	25 (47.2%)	0	0	0
Grade 3	6 (11.3%)	0	0	0

## Discussion

This study gives an overview of EC observed in a single medical centre over six-year period. All the cases seen during those periods were endometrioid carcinoma while uterine serous carcinoma or clear cell carcinoma which is the prototypic type II tumour was not encountered in the medical centre. The incidence of this more aggressive uterine serous carcinoma is nonetheless rarer as compared to endometroid carcinoma, where it accounts for only 10% of EC while clear cell carcinoma is even less common [11]. Most of the patients diagnosed with EC in this study were from Malay ethnicity, followed by Chinese and Indian which represent the cohort of patient seen in the medical centre of study. As a comparison, data from National Cancer Registry Malaysia (2007-2011) shows that the incidence of EC was the highest among Chinese, followed by Malay and Indian [3].

Most of the cases in this study were low-grade tumour (grade 1 and 2) that behaviorally is less aggressive and associated with better outcome and prognosis. A total of 83.1% of the cases in this study were diagnosed in early stage (stage I and II). There is no established guideline used for HER2 testing in endometrial carcinoma [13]. In this study, the interpretation of the staining is based on ASCO/CAP scoring system for breast cancer. HER2 has been reported to be overexpressed by immunohistochemistry in <10% of endometrial cancer, out of which 26% of serous carcinoma showed strong expression (score 3+) [14]. An earlier study in 1999 had shown the overexpression of HER2 protein in 17% of endometrial carcinoma. However, HER2 gene amplification was only proved in 21% of those cases, and this was associated with higher carcinoma grade especially the clear cell and serous subtype [15].

A similar observation was reported in 2016, in which HER2 overexpression and gene amplification were found to be associated with high grade and high stage endometrial cancer. The highest rate of expression was seen in serous carcinoma (which showed 43% of HER2 protein overexpression and 29% gene amplification). The authors also noted that only 3% of HER2 protein overexpression and 1% gene amplification exhibited in grade 1 endometrioid carcinoma which was the lowest rate seen among other types of EC [16]. These factors may explain the lack of HER2 protein expression seen in the cases of this study, the majority of which (47 out of 53 cases) were comprised of lower grade EC. Cases of serous or clear cell carcinoma were not encountered during the period of study to fully assess the expression of HER2 in high grade prototypical type II EC. Nevertheless, HER2 may still play a significant role in prognosis. It could also be one of the potential therapeutic targets in advanced cases as well in high grade endometroid carcinoma [11].

Overexpression of HER2 has been associated with higher grade and stage that have been linked to poor prognosis of EC. A study in 2014 has reported HER2 positive expression in 14 out of 77 cases of EC (18.2%). The rate of positivity was significantly increased in patients with higher FIGO stage (p<0.001) [17]. Whereas, in another study in the same year which focused on hormone-dependent endometrial carcinoma had shown that HER2 positivity with negative hormone receptors status - oestrogen (ER) and progesterone (PR) were correlated with poorer outcome. Meanwhile, the opposite expression (ER positive, PR positive and HER2 negative) was associated with more favourable prognosis (p = 0.002) [18].

HER2 overexpression has become the focus of several studies as it provides the scientific basis for targeted immunotherapy. Overexpression of HER2 has a favourable clinical response to trastuzumab, a humanized monoclonal immunoglobulin (Ig) G1 antibody against HER2. Trastuzumab is currently approved by Food and Drug Administration (FDA) in the treatment of HER2-overexpressing breast cancer and metastatic gastric or gastroesophageal junction adenocarcinoma [11]. In vitro studies have demonstrated that trastuzumab results in antibody-dependent cellular cytotoxicity in the range of 25% to 60% against HER2 overexpressing uterine serous carcinoma. However, no significant activity was observed in advanced or recurrent EC patient as evidenced by Fleming *et al*. [19].

Intratumoral heterogeneity may account for negative IHC results observed in the current study as only one section of the tumour specimen was immunostained for HER2. Heterogeneity in HER2 protein expression in gynecological malignancy from ovary and endometrium was reported to be less than 2% [20]. A higher percentage of tumour heterogeneity was noted by Buza *et al*. in 2013 who has observed a significant tumour heterogeneity in HER2 protein expression in 20 out of 38 cases (53%) of endometrial serous carcinoma [13]. Another similar study in the same year that evaluated 17 cases of endometrial serous carcinoma with heterogenous HER protein expression had found clusters gene amplification in 72% of the cases. The authors concluded that the current HER2 guideline used for breast cancer may not be appropriate for interpretation of HER2 expression in endometrial serous carcinoma [21].

HER2 gene amplification can be confirmed by in-situ-hybridization study (ISH). Discordant result may be observed, in which positive-HER2 protein cases in immunohistochemical study (IHC) showing negative result on ISH study. The percentage of HER2 protein expression and gene amplification may differ between various histologic grade of endometrial carcinoma. Morrison *et al*. had observed higher HER2 protein expression and gene amplification in grade 3 cancer (31% and 15%) as compared to grade 2 (7% and 3%) and grade 1 cancer (3% and 1%), respectively [16].

The most common causes of false-negative immuno-staining are poor tissue fixation, over-diluted or improperly optimized antibodies and non-optimized epitope retrieval method [22]. In this study, as per routine laboratory protocol, all the specimens have been fixed in 10% formalin for 24 hours prior to sampling with bivalving of the uterus was performed to ensure better penetration of formalin to tumour tissue. Heat-mediated antigen retrieval with a temperature of 110°C was used. The primary antibody was optimized well before the same protocol was followed in staining of the samples. The control tissue was working well using the same method.

Other factors that may affect immunohisto chemical results include degeneration of epitope that may occur with longer duration of tissue block storage. A study by Manne *et al*. evaluating the effects of the duration of paraffin block storage (ranged from three to 16 years) on immunohistochemistry result, had found no significant decline in the intensity of immunohistochemical stains. They concluded that long term storage of paraffin blocks does not prevent the usage of archival tissue to determine the prognostic or diagnostic importance of biomarkers by immunohistochemistry test [23]. However, an earlier study had shown a significant loss of antigenicity in paraffin tissue blocks stored for more than 12 weeks impacting the immunohistochemistry result [24]. The tissue blocks from this study have been in the archive for one to six years before immunohistochemical staining was performed. Nonetheless, all cases showed lack of HER2 expression which may exclude this factor as contributing to the negative protein expression seen in the study.

## Conclusion

This study showed that there is no significant HER2 expression in endometroid type of endometrial carcinoma. However, a follow-up study with a larger number of samples from different types of endometrial carcinoma is useful to fully evaluate HER2 expression and to correlate it with clinicopathologic parameters. Testing of several tumour tissues blocks to assess larger tumour area to exclude possible tumour heterogeneity, as well as correlation with HER2 gene amplification status by in-situ-hybridisation, are also recommended.

### What is known about this topic

- Endometrial carcinoma is a common cancer worldwide contributing to significant morbidity and mortality;
- HER2 protein overexpression is reported in high stage and high grade of endometrial carcinoma therefore possible to be one of therapeutic target in patient’s management.

### What this study adds

- There is insufficient evidence to support the routine usage of HER2 immunohisto chemical staining in endometrial carcinoma specifically the endometrioid type;
- There is insufficient evidence to support HER2 as a significant therapeutic target in endometrioid endometrial carcinoma.
